# Supplementary material for: Development of a culture-independent whole-genome sequencing of Nipah virus using the MinION Oxford Nanopore platform
Source: Microbiol Spectr. 2025 Apr 16;13(6):e02492-24. doi: 10.1128/spectrum.02492-24 (PMC12131749; doi:10.1128/spectrum.02492-24)
Supplement: Figure S1 — Read length by the average read quality for the Nipah virus whole-genome sequencing from 12 samples with an Oxford Nanopore MinION flow cell. [file spectrum.02492-24-s0001.pdf]

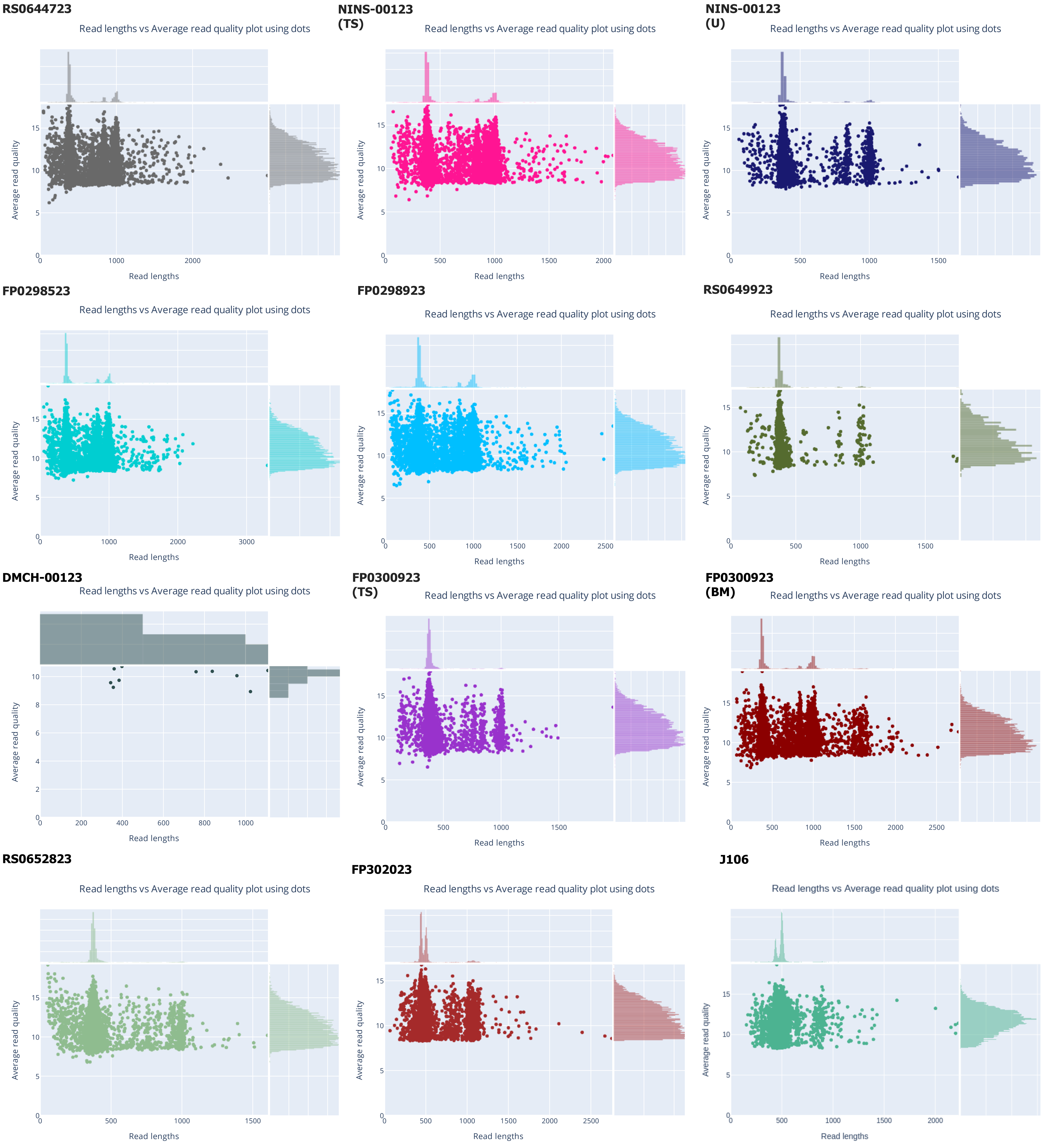

Supplementary Figure 1: Read length by average read quality for Nipah virus whole genome sequencing from 12 samples with an Oxford Nanopore MinION flow cell;
