# Supplementary material for: Development of a culture-independent whole-genome sequencing of Nipah virus using the MinION Oxford Nanopore platform
Source: Microbiol Spectr. 2025 Apr 16;13(6):e02492-24. doi: 10.1128/spectrum.02492-24 (PMC12131749; doi:10.1128/spectrum.02492-24)
Supplement: Figure S2 — Scatter plot showing mean read quality (Q) as a function of the Ct value for 12 NiV samples. [file spectrum.02492-24-s0002.pdf]

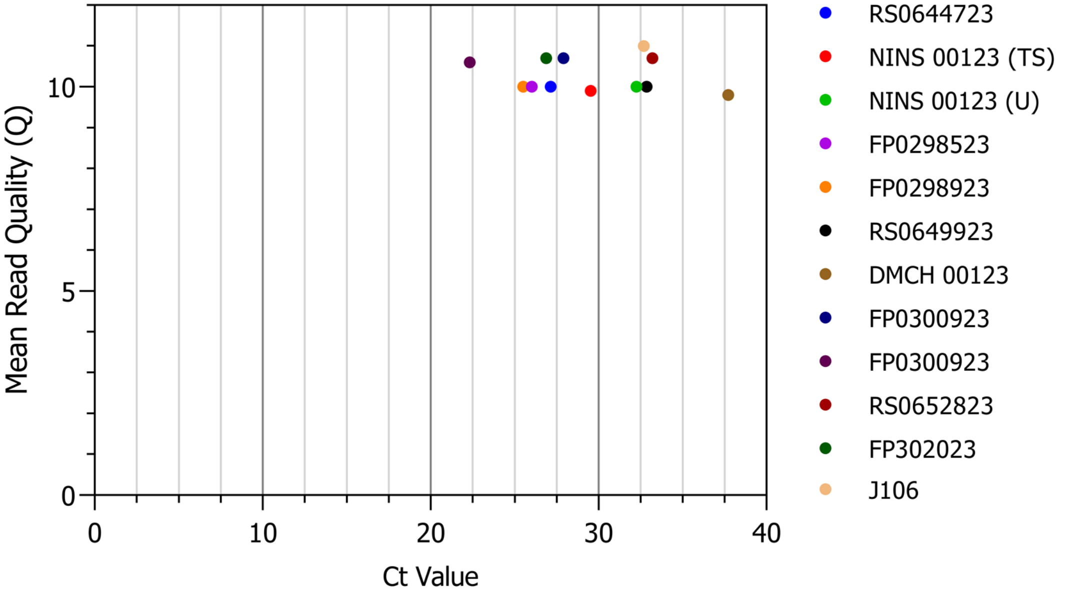

Supplementary Figure 2: Scatter plot showing Mean Read Quality (Q) as function of Ct value for 12 NiV samples
