# Supplementary material for: Development of a culture-independent whole-genome sequencing of Nipah virus using the MinION Oxford Nanopore platform
Source: Microbiol Spectr. 2025 Apr 16;13(6):e02492-24. doi: 10.1128/spectrum.02492-24 (PMC12131749; doi:10.1128/spectrum.02492-24)
Supplement: Table S1 — Original and modified ARTIC primer sets used in this study. [file spectrum.02492-24-s0003.pdf]

Supplementary Table 1: The original (left) and modified (right) ARTIC primer sets, with the modified bases highlighted in bold red, were used in this study.

Sl No	Primer	Original ARTIC Primer set for NiV	Modified ARTIC primer set for NiV	Pool
		Primer Sequence (5'-3')	Primer Sequence (5'-3')	
1	NiV_BGD_1_LEFT	CCAAACAAGGGAGAATATGGATACGT	CCAAACAAGGGA**A**AATATGGATACGT	1
2	NiV_BGD_2_LEFT	GTTGGAGCTGCTTTCACACTCA	GTTGGAGCTGCTTT**T**ACACTCA	2
3	NiV_BGD_3_LEFT	GCTAAAGCAGTTACAGCTCCCG	GC**C**AAAGCAGTTACAGCTCCCG	1
4	NiV_BGD_4_LEFT	AGGTATCCAGCACTTGCACTCA	AGGTATCCAGCACTTGCACTCA	2
5	NiV_BGD_5_LEFT	GAACATGGCAAATAGACTGGGACT	GAACATGGC**G**AATAGACTGGGACT	1
6	NiV_BGD_6_LEFT	CCTCAGATCAAGACTGGCTGCA	CCTCAGATCAAG**G**CTGGCTGC**T**	2
7	NiV_BGD_7_LEFT	ATGGGCAAACCGAAGAACCAAT	ATGGGCAAA**G**CGAA**C**AACCAAT	1
8	NiV_BGD_8_LEFT	CACTTAACTTGACCAAGGTCTACCA	CACTTAACTTGACCAAG**A**TCTAC**T**A	2
9	NiV_BGD_9_LEFT	CAGAAGACATACGGACGATCAAGT	CAGAAGACATACGGACGATCAAG**C**	1
10	NiV_BGD_10_LEFT	CCTGAGAGAGGGTGGAGTGATT	CCTGAGAGAGGGTGGAGTGAT**C**	2
11	NiV_BGD_11_LEFT	TTGTACACATCAGATGACGAAGAGG	TTGTACACATCAGATGACGAAGAGG	1
12	NiV_BGD_12_LEFT	AGCTGCTGAAGGAGAACTCACT	AG**T**TGCTGAA**A**GAGAA**T**TCA**T**T	2
13	NiV_BGD_13_LEFT	AGAAGGCAAGAGTGTCAATGCG	AGAAGGCAAGAGTGTCAATGCG	1
14	NiV_BGD_14_LEFT	ACATTGAAGAACAAGTTAAAGAGATCCCA	ACATTGAAGAACAAGTTAAAGAGATCCCA	2
15	NiV_BGD_15_LEFT	TATGGGGCAGCTGTACAGTTGA	TATGGGGCAGCTGT**C**CA**A**TTGA	1
16	NiV_BGD_16_LEFT	ACAATCTCCCACAAGTAGACAATGG	ACAATCT**T**C**Y**ACA**T**GTAGACA**G**T**R**G	2
17	NiV_BGD_17_LEFT	ACAATGATGTCAACTTTCATACCTAAACA	ACA**G**TGATGTCAACTTTCATACATAA**GT**A	1
18	NiV_BGD_18_LEFT	TTGGGAGCATGGTGGGTATCTT	TTGGGAG**A**ATGGTGGGTATCTT	2
19	NiV_BGD_19_LEFT	TCTGGCCCTCTAAATCACCTCG	TCTGG**A**CCTCTAAATCACCT**T**G	1
20	NiV_BGD_20_LEFT	GCTTCAAGGTTGCCTCCTTCAT	GCTTCAA**A**GTTGC**T**TCCTTCAT	2
21	NiV_BGD_21_LEFT	TGTGTTGCAGCCTTCTATTCCA	TGTGTTGCAGCCTTCT**G**TTCC**G**	1
22	NiV_BGD_22_LEFT	ACAAGCATTCTTACCATTGGATCAAC	ACAAGCATT**A**TTAC**T**AT**CT**GATCAAC	2
23	NiV_BGD_23_LEFT	TCTCGGAGTGTAGTGTTGGGAT	TCTCGGAGTG**C**AGTGT**C**GGGAT	1
24	NiV_BGD_24_LEFT	CAGCTCAAATCACTGCAGGTGT	CAGCTCAAAT**T**ACTGCAGGTGT	2
25	NiV_BGD_25_LEFT	TCTCAGGCATTCGGTGGAAATT	TCTCAGGCATTCGGTGGAAATT	1
26	NiV_BGD_26_LEFT	GAGCGTGATCTGCAACCAAGAT	GAG**T**GTGATCTGCAACCAAGAT	2
27	NiV_BGD_27_LEFT	TCAGCTTAGGGAAATATCTGGGGT	TCAGCTT**R**GG**A**AAATATCT**T**GGGT	1
28	NiV_BGD_28_LEFT	GGAGAGTCAGACCTACAAGCAGT	GGAGAGTCAGACCTACAAG**T**AGT	2
29	NiV_BGD_29_LEFT	ACACGAGCGCTAACCTATACACT	**G**CA**A**GAGC**T**CTAA**T**CTATACACT	1
30	NiV_BGD_30_LEFT	TCCTATAATCACATCATAGGAGTGAACCA	T**T**CT**T**TAATCACAT**T**ATAGGAGTGA**G**CCA	2

31	NiV_BGD_31_LEFT	TCCAAAATTACACAAGATCAACAGACAA	TCCAAAACTACACAAGATCAACAGATAA	1
32	NiV_BGD_32_LEFT	ACAGACAGAAGGGGTGAGCAAT	GCAGACAGAAGGAGTGAGCAAT	2
33	NiV_BGD_33_LEFT	CTGGACCCCACCAAATCCAAAC	CTGGACCCCATCAAATCCAAAC	1
34	NiV_BGD_34_LEFT	TGATTCAAATTGTCCCATCACGAAG	TGATTCAAATTGTCCCATCGCAGAR	2
35	NiV_BGD_35_LEFT	TAACACGGTAATATCAAGACCCGG	CAACACGGTAATCTCAAGACCTGG	1
36	NiV_BGD_36_LEFT	TGGTTGAGATATATGACACAGGAGACA	TGGTTGAGATATACGACACAGGAGACA	2
37	NiV_BGD_37_LEFT	TCTTGATCGGTGATCTTTGAGAACA	TCTTGATCAATGATCTTTGAAAATA	1
38	NiV_BGD_38_LEFT	ACCCAGGTCCTTGATTATGCCA	ACCCAGGTCCTTGATTGTGCTA	2
39	NiV_BGD_39_LEFT	CAGCCCAGTGATGATAAAAGACTGT	CAACCTAGTGATGACAAAAGACTGT	1
40	NiV_BGD_40_LEFT	TGGGATGCTGCAAAATATCACTAGA	TGGAATGCTACAAAAYATCACTAGA	2
41	NiV_BGD_41_LEFT	TCTTACACCCGAAATGGTTCTAATGT	TCTTACACCCGAAATGGTTCTAATGT	1
42	NiV_BGD_42_LEFT	TGAGTGAGTGTGGTTTTACAGATCA	TGAGTGAGTGTGGCTTTACAGAYCA	2
43	NiV_BGD_43_LEFT	TGGGGAATCTTTGACCATTGATGAC	TGGGGAATCTTTAACCATTGATGAT	1
44	NiV_BGD_44_LEFT	GCTTGAAGGAGAAAGAGACGAAGC	GTTTGAAGGAGAAAGAGACAAAGC	2
45	NiV_BGD_45_LEFT	TCTGCTCTCAATAATCCGTGCC	TCTGCTCTCAATAATCCGTGCC	1
46	NiV_BGD_46_LEFT	TGGATGCACAAACGACTAGAAAGA	TGGATGCATAAACGACTAGAAAGG	2
47	NiV_BGD_47_LEFT	AGATCTGTGCAAAGCAAGCTCA	AGATCTGTGCAAAACAAGCTCA	1
48	NiV_BGD_48_LEFT	CAGCAGCTTCTCATATCAACTGAGT	CAGCAACTCCTTATATCAACTGAGT	2
49	NiV_BGD_49_LEFT	TGCCTGACTCACAAAGCATCAC	TGCCTGACTCGCAAAGTATTAC	1
50	NiV_BGD_50_LEFT	AGTGGACTTCAGCCAAAGTTAGTT	AGTGGAATTCAGCCAAGGTTAGTT	2
51	NiV_BGD_51_LEFT	TGGGTGGTTCTTTGTACCTAGGG	TGGGTGGTTTTTTGTACCTAGGG	1
52	NiV_BGD_52_LEFT	TGCAGGGAGTGTACTCAACAGA	CGCAGGGAGTGTACTCAACAGG	2
53	NiV_BGD_53_LEFT	TGATCCAGACCCCGTTTCAGAA	TGATCCAGATCCTGTTTCAGAA	1
54	NiV_BGD_54_LEFT	AGGAAGACATACTATGGTCGACCT	AGGAAGACATACTATGGTTGATCT	2
55	NiV_BGD_55_LEFT	ACATCCAAACATCTCTGTGTTATAATTGAC	ACATCCAAACATCTCTGCGTTATAATTGAC	1
56	NiV_BGD_56_LEFT	ACAGGAGTGTTAGACATCAGAGGT	ACAGGGGTGTTAGATATCAGAGGT	2
57	NiV_BGD_57_LEFT	GGTCAAAGAGAACTGAAACTATTTCCCT	GGTCAGAGAGAACTGAAGCTATTYCCAT	1
58	NiV_BGD_58_LEFT	TAGCATACACCCCTGGATTCCC	TAGCATACACCCCCGGATTCCC	2
59	NiV_BGD_59_LEFT	CAAGTACTTCTCCAAGCAGGGC	CAAGTACTTCTCCAAGCAGGGC	1
60	NiV_BGD_60_LEFT	ACAGATCACCTTCACACCACCT	ACAGGTCGCCTTCATACCATTT	2
61	NiV_BGD_1_RIGHT	TGTCTGGGTCATTGAGGAGACTT	TGTCTGGGTCATTGAGGAGACTT	1
62	NiV_BGD_2_RIGHT	GGATTGACTCTCTTTTGTTGGACG	GGATTGACTCTCTTTTGTTGAACG	2
63	NiV_BGD_3_RIGHT	GGCCAATTTCTCTGTAGAGTAGCA	GGCCAATTTCTCTGTAGAGTAGCA	1
64	NiV_BGD_4_RIGHT	ACTCTCTTGCCTTCCTGCTGAT	ACTCTCTTGCCTTCCTGCTGAT	2

65	NiV_BGD_5_RIGHT	TTCCCCTTGAGTTCTGTTGCTG	TTCCCTTTGATCTTTGTTGCTG	1
66	NiV_BGD_6_RIGHT	ACACTAATAGTTCAAGATTTTGAGCAAGT	GCACTAATAGCTCAAGATTTTGAGTAAAT	2
67	NiV_BGD_7_RIGHT	AGATTGCAGTTCACTTGTGCGA	AGATTGCAGTTCATTTATGCGA	1
68	NiV_BGD_8_RIGHT	GTGCACTGCAGAAAATCTTCCC	GTGCACTGCAGAAAATCCTCCC	2
69	NiV_BGD_9_RIGHT	ACAAGACATACATTCCCATTGTTTGC	ACAAGACATACATCCCCATTGTTTGC	1
70	NiV_BGD_10_RIGHT	TGAGCTTCCTGCAAACTCATCTT	TGAACTCCCTGCAAACTCATCCT	2
71	NiV_BGD_11_RIGHT	GGAGGCTGAGCATCTTTCCCTT	AGAGGCTGGGCATCTTTCCCTT	1
72	NiV_BGD_12_RIGHT	TCGTTGTCGTCTACGGGGTTTA	TCGTTGTCATCTGCAGGGTTTG	2
73	NiV_BGD_13_RIGHT	CCTTCAATGGTTGAGAGTGCGG	CCTTCAATAGTTGAGAGTGCAG	1
74	NiV_BGD_14_RIGHT	TCTGGATGAATCATCTGCCATAGG	TCTGGACGAATCATCTGCCACGGG	2
75	NiV_BGD_15_RIGHT	TGTGTTATCGTCTGAGGGCTGG	TGTGTTATCGCCCGAGAGCTAG	1
76	NiV_BGD_16_RIGHT	TGCGTGAGAGATCTACAATACAAATCG	TGCGTGAATGAACTACAATACGAATCG	2
77	NiV_BGD_17_RIGHT	AGCTCCTGGGGTATAGATCTTGT	AGCTCCTGGGGTGTAGATCTTGT	1
78	NiV_BGD_18_RIGHT	TCAACGTTCCGACAAACCTTGA	TCAACGTTTCGGCAGACCTTAA	2
79	NiV_BGD_19_RIGHT	TGCAATTTCATCCTATCAATCTTCCTCC	TGCAATTTCATCCTATCAATCTTTCTCC	1
80	NiV_BGD_20_RIGHT	TTCTGTTTAGCCCTTTAGAATTCTCCC	TTCTACTTAGCCCTTTAGAATTCTCCC	2
81	NiV_BGD_21_RIGHT	AAACCAGGAATTTCTTTGCCTTTCA	AAATCTAGAATCTCTTTGTCTTTCG	1
82	NiV_BGD_22_RIGHT	TCTTGTTACTCCTTTGACAAGTCCA	TCTTGTTATTCCTTTGACAAGCCCA	2
83	NiV_BGD_23_RIGHT	TTTGAGTTTGTTGATGTTGTCAGCA	TTTGAGTTTGTTGATGTTGTCAGCA	1
84	NiV_BGD_24_RIGHT	GTCTTCTGTAGCGTAACCCAATGT	GTCTTCTGTAGCGTAACCCAACGT	2
85	NiV_BGD_25_RIGHT	AGTCGATCCCGTTAAACATTCTCTC	AGTCGACCCCGTCAAACATTCCCTC	1
86	NiV_BGD_26_RIGHT	AGGACTGATTCATGCTGGATATCTG	AGGACTGATTCATGCTAGATATTTG	2
87	NiV_BGD_27_RIGHT	TTCATCAATCTGAATACACTATGTCCCA	TCAATCAATCTGAATACACTACGTCCCA	1
88	NiV_BGD_28_RIGHT	ATTTTCTTGGTCGAAATGCTATTGTAGT	ATTTCCTTGGCTGAGATGTTGTTATAGT	2
89	NiV_BGD_29_RIGHT	ACGTCAACAATACTTTCCTTCCCC	GTGCCAGCAATGCTTTCTTCTCTT	1
90	NiV_BGD_30_RIGHT	GGATACCCTGCAACGCATCTTT	GGATACCTCGCAATGCATCTTT	2
91	NiV_BGD_31_RIGHT	TCTGATTAGATGTCTTTTGCAGGCA	TCTGATTAGATGTCTTTTGCAGACA	1
92	NiV_BGD_32_RIGHT	AGGGTCTCCAACAGTTGACACT	AGGGTCTCCAACAACTGACACT	2
93	NiV_BGD_33_RIGHT	TCTAATCCCCATAGATAGCCTGCA	TCTAATCCCCATAGATAGCCTGCA	1
94	NiV_BGD_34_RIGHT	AACTCCTTCCCAGCAGATCTCT	AACCCCTTCCCAGCAAACCTCT	2
95	NiV_BGD_35_RIGHT	ATGAGGTTGATTTTTATGTACATTGCTCT	ATGAGATTAACTTTTATGTACATTGCTCT	1
96	NiV_BGD_36_RIGHT	TCGGGCCGAAAATACTAAGTCTC	CCAAATCGAATATATTAAGTCCC	2
97	NiV_BGD_37_RIGHT	GGTAACAACGCTTTCAGTCTATGGT	GGTAATAACGTCTTCAGTCTGTAAG	1
98	NiV_BGD_38_RIGHT	TTTATGTTGTTTCTAATGTAATCACCCTGT	TTAACGTTATTTCTGATATAATTACCTTGC	2

99	NiV_BGD_39_RIGHT	ACCATTTAGATTGACTCATATTCTTCCCT	ACCATTTAGATTGACTCATATTCTTCCCT	1
100	NiV_BGD_40_RIGHT	TCCGATTTGACTGTTGTCTCCATC	TCTGATTTGATTGTTGTCTCCATC	2
101	NiV_BGD_41_RIGHT	ACGAAAGAAAGAAAAGAACTCTGCC	ACGAAAGAAAGAAAAGAACTCTGCC	1
102	NiV_BGD_42_RIGHT	TTGAATCCCACAGAATGATTCCCA	TTGAATCCCACAGAATGATTCCCA	2
103	NiV_BGD_43_RIGHT	TGCACGCATTTTGTAGGTCATCT	TGCACGCATCTTGTAGGTCATCT	1
104	NiV_BGD_44_RIGHT	TCGTGTATTGGGTGACATGTTATGA	TCGTATGTTGGGTGACATGTTATGA	2
105	NiV_BGD_45_RIGHT	GGGCAATTAGGGTCTGCAACAT	GGGCAATTAGGATCCGCAACAT	1
106	NiV_BGD_46_RIGHT	TGTAGCTTTAAGATTGTGGCCGA	GGTAGCTTTAAGGTTGTGGCCGA	2
107	NiV_BGD_47_RIGHT	AATGGGAGATGTCACATCCAGTG	AATGGGAGATGTCACATCCAGTG	1
108	NiV_BGD_48_RIGHT	TTGGGTTCGGTGAGTTCCTCAG	TTGGATTCGGTGAGTTCCTCAG	2
109	NiV_BGD_49_RIGHT	TTGATGATATCAAGTCATTTTGTCTTCTGT	TTGATGATATCAAATCATTCTGTTTTCTGT	1
110	NiV_BGD_50_RIGHT	TCCGATCTTTCATCCGTACTTGATC	TCTGATCTCTCATCTGTACTTGATC	2
111	NiV_BGD_51_RIGHT	TCTCCCTCAATTCTGAAATCTAGATTGT	TCCCCCTCAATTCTGAAATCCAGATTAT	1
112	NiV_BGD_52_RIGHT	TTCCTCAGTTGACCATAAAGGAAAGT	CTCCTCAGTTGACCATAAAGGGAAGT	2
113	NiV_BGD_53_RIGHT	AGGATGTGACAAGGCATTTGACA	AGGATGTGACAAGGCGTTTGACA	1
114	NiV_BGD_54_RIGHT	TGTGGGTTTAGATCTATTATCCAAGGAG	TGTGGGTTCAGATCTATTATCCAAGGAG	2
115	NiV_BGD_55_RIGHT	TGCTGATTTTGTGTTCATAGACAGGA	CGATGATTTCGTGTTCATAGACAGGA	1
116	NiV_BGD_56_RIGHT	CGTCAGAGATGGGTCTTCCTCA	CGCCAAAGATGGGTCTTCCTCA	2
117	NiV_BGD_57_RIGHT	GGGAAACACACCAGTACTAGTCC	GGGAAACACACCAGTACTAGTCC	1
118	NiV_BGD_58_RIGHT	TTCACTCTTAAGAATTTCTGGCCCA	TTCACTCTTAAGGATTTCTGGCCCA	2
119	NiV_BGD_59_RIGHT	CTTCTCTCTTGCATCCCTTTAGGT	CTTCTCTCTTGCATGCCTTTAGGT	1
120	NiV_BGD_60_RIGHT	ACCTTAGATCAACATAAGTTATCAGTTGGT	ACTTTGGACCAGCATAAATTGTCGGTCGGT	2
